# Phylogenetic Relationships of the Mangalitsa Swine Breed Inferred from Mitochondrial DNA Variation

**DOI:** 10.3390/ijms13078467

**Published:** 2012-07-09

**Authors:** Sergiu Emil Georgescu, Maria Adina Manea, Andreea Dudu, Marieta Costache

**Affiliations:** Department of Biochemistry and Molecular Biology, Faculty of Biology, University of Bucharest, Splaiul Independentei 91-95, Bucharest 050095, Romania; E-Mails: georgescu_se@yahoo.com (G.S.E.); adina_manea@yahoo.com (M.M.A.); tn_andreea@yahoo.com (D.A.)

**Keywords:** mangalitsa pig, primitive breed, mitochondrial DNA, phylogeny

## Abstract

The Mangalitsa pig, a swine breed belonging to the protected gene fund of original and primitive animal breeds of the FAO (Food and Agriculture Organization), has been known to inhabit Romanian territories since the 19th century. The aim of this study was to compare the Mangalitsa breed with several European and Asiatic swine breeds in order to emphasize its uniqueness and to elucidate its origin. For this purpose, we analyzed a 613 bp mitochondrial DNA D-loop fragment and 1140 bp of the cytochrome b gene in a population of Mangalitsa pigs and the polymorphic sites were compared with sequences from GenBank originating from other swine breeds. Taking into account the total of 24 breeds and 5 different Wild Boar populations analyzed, 86 polymorphic sites representing 32 haplotypes were observed, with an average percentage of polymorphic sites of 4.9%. Three Neighbor-Joining phylogenetic trees were constructed based on Kimura 2-parameter distances, using D-loop, cytochrome b and mitochondrial reunited sequences. For the analyzed Mangalitsa population, four distinct haplotypes were identified, including one that was common to other breeds. Our study suggests that the Mangalitsa swine originate from primitive breeds which might be directly derived from the Wild Boar.

## 1. Introduction

In the last years, the conservation of animal and plant biodiversity has become a major international goal in environmental sciences. As a result, maintaining the biodiversity of local breeds of domestic animals, especially the ones of economic interest, has become a priority.

At the international level, there is an increasing concern regarding the study and genetic characterization of local populations, the so-called rare animal breeds. Most of the research performed to date refers to the breeds’ genetic characterization and the assessment of the phylogenetic relations among them. During recent years, a series of studies were performed regarding local horse breeds [1–3], bovines [4,5], swine [6–10] and sheep [11,12]. The genealogic analysis of these breeds provided valuable information regarding the current status of unimproved specimens. The characterization of the genetic variability of local breeds is, currently, one of the priorities of scientific research in animal genetics, as it is dictated by the re-assessment of practices in livestock breeding as well as by the conservation of genetic resources.

The Mangalitsa breed is considered to be a direct descendant of the Wild Boar and is part of the European primitive breeds that have not been ameliorated by crossbreeding with other swine breeds. By contrast to the swine breeds specialized in meat production, the Mangalitsa breed is specialized in lard production. Nevertheless, apart from excess fat, swine from this breed also produce interstratified meat of superior quality.

According to FAO (Food and Agriculture Organization) the Mangalitsa breed originates from the current territory of Hungary and was officially acknowledged in 1927. To be more specific, the Mangalitsa breed originates from the Balkan region and is a result of the cross-breeding of individuals from the Sumadija breed from Serbia with the Bakonyi and Szalontai breeds from the territory of the Austro-Hungarian Empire which took place in in the 19th century [13]. Following the steep decline in the number of specimens after the Second World War, the Mangalitsa breed is now classified as an endangered breed, on the brink of extinction, with only three varieties surviving to date (*i.e*., Blonde, Swallow-Bellied and Red), which can be distinguished by fur colour [13].

The Mangalitsa is currently found only on the territory of the former Austro-Hungarian Empire. These pigs have woolly coat, lop ears and are adapted to adverse conditions of feeding and management. Due to the low meat production and reduced prolificacy, the Mangalitsa breed has been slowly but gradually replaced. Presently, it is the only European swine breed whose body is entirely covered with thick fur, consisting of long and curly hairs. Conversely, only two centuries ago, such primitive breeds could have been encountered in the Mediterranean basin and in the entire Balkan region [14]. However, these breeds have been lost largely due to their replacement by modern, improved breeds, with a much higher production of meat.

The mitochondrial genome of vertebrates presents some useful features such as maternal transmission, higher rate of mutation in comparison to the nuclear DNA, rapid evolution and lack of recombination; consequently, these characteristics recommend it as a useful tool for phylogenetics studies. Past studies have already determined the complete sequence of the pig mitochondrial genome and the organization of mitochondrial genes [15]. The mitochondrial genome has a size of 16,613 bp and, with the exception of the D-loop region, consists only of structural genes without any non-encoding bases. In order to investigate specific mitochondrial DNA (mtDNA) phylogenetic markers, sequence specific primers were designed flanking these markers. Amplified fragments were sequenced, and the results were analysed using molecular phylogeny specific software. These fragments can be analyzed in terms of conservation by comparison to other populations and will serve for the construction of phylogenetic trees.

The aim of this study was to compare the Mangalitsa breed with various swine breeds with distinct geographical distribution in order to assign their origins. The comparative analysis was performed at a molecular level by investigating a fragment from the D-loop mitochondrial region and the cytochrome b gene. Different haplotypes obtained for the Romanian Mangalitsa individuals were compared with haplotypes from different European and Asian breeds.

## 2. Results and Discussion

In this study, partial sequences of 613 bp from the D-loop control region and 1140 bp from the cytochrome b gene were determined for all the sampled individuals (45 individuals from the Mangalitsa breed and 15 Wild Boars from Romania). Four distinct haplotypes were identified among the 45 sequences from Romanian Mangalitsa, while only one haplotype was identified for the 15 sequences from Romanian Wild Boar.

For an overview of the phylogenetic information content, our sequences representing different haplotypes were aligned and compared with sequences from other European and Asian swine breeds available from GenBank. This analysis showed 86 variable sites (75 transitions, 10 transversions and one deletion), representing 4.9% from the total number of nucleotides.

**Figure 1:**
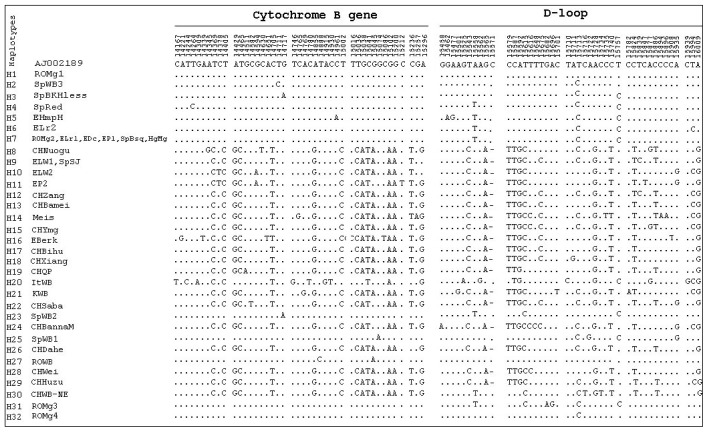
Haplotypes and variable sites in the D-loop region (between nucleotides 15,455 and 16,068) and cytochrome b gene in 24 swine breeds, 5 Wild Boar populations and the reference sequence (GenBank AJ002189). The numbers represent the position occupied in the sequence. Identical sites are indicated by the symbol “·” and the deletions with “−”.

For the animal specimens we have analyzed in our study, at the level of the cytochrome b gene we identified 43 single-nucleotide polymorphisms from which 38 are transitions and 5 are transversions: A→T (14,339, 14,651); T→G (14,746); C→G (14,765) and C→A (14,961). Regarding the D-loop region, 43 single-nucleotide polymorphisms were identified, including 37 transitions, 5 transversions (A→T, 15,558; C→A, 15,782, 15,894 and 15,896; C→G, 15,939) and one deletion at position 15571. A total number of 32 different haplotypes was identified in all pig populations. From the 32 haplotypes, five represent haplotypes discovered by us in Romanian Mangalitsa breed (ROMg1, ROMg2, ROMg3 and ROMg4) and Romanian Wild Boar (ROWB). The polymorphic sites observed per each breed are shown in Table 1 and the results obtained in terms of nucleotide variation and haplotypes are presented in Figure 1.

Among the 32 distinct haplotypes, two were common for various breeds: the H7 haplotype is common in the Mangalitsa breed in Romania, Hungary, Landrace, Duroc and Basque, while the H9 haplotype is common in the Large White and Spotted Black Jagubo from Spain. Four haplotypes were identified in 45 individuals of Romanian Mangalitsa with haplotype diversity (H_d_) 0.755, nucleotide diversity (Pi) 0.00158, Theta (per sequence) Eta 1.37214 and Theta (per site) Eta 0.00078. One of these haplotypes (ROMg2) is common in other breeds, as well as in the Mangalitsa breed from Hungary. Moreover, for the Romanian Wild Boar there is only one distinct haplotype (H27).

By using the “Kimura 2-parameter” algorithm [16], the genetic distances as well as their standard deviations were calculated. Based on these results, the phylogenetic relationships among swine breeds were inferred by the Neighbor-Joining algorithm. We constructed three phylogenetic trees: two of them were based on the cytochrome b gene and on the partial sequence of the D-loop region, respectively, while the third tree was built using both mitochondrial markers.

**Figure 2:**
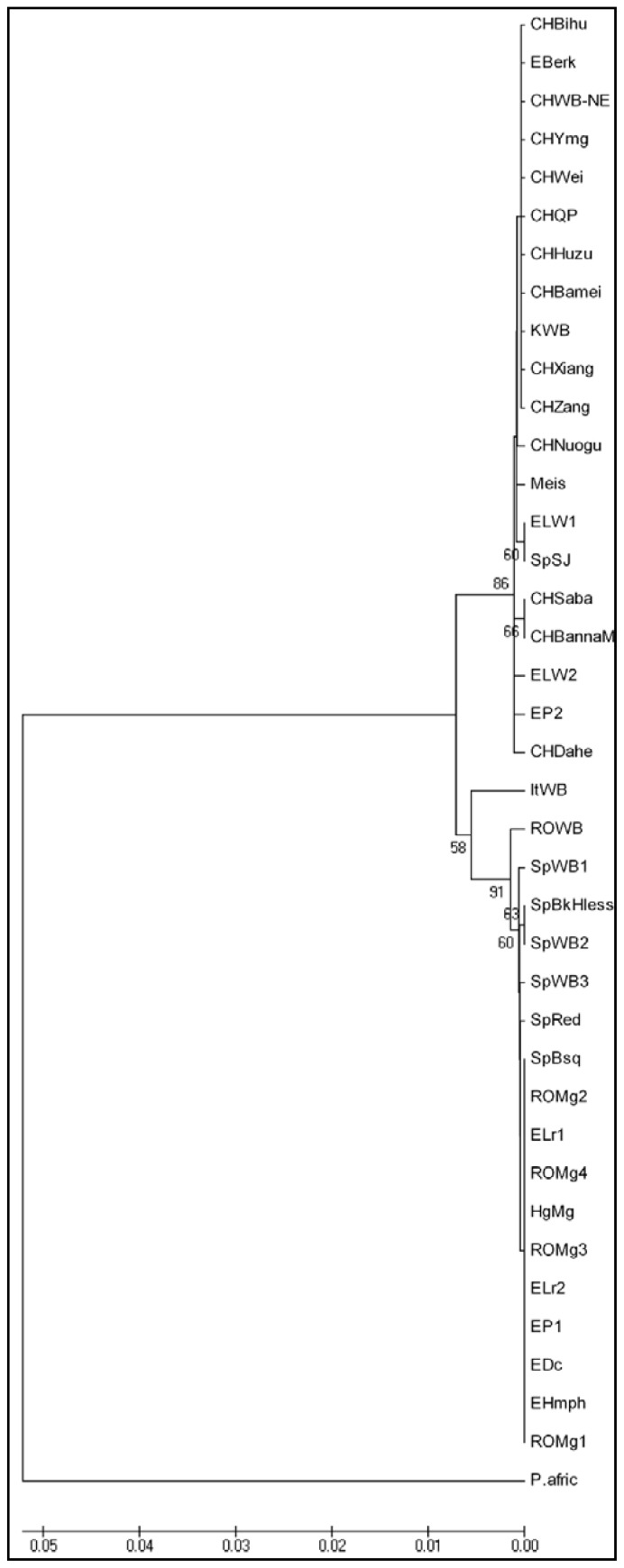
Neighbor-Joining tree based on cytochrome b sequence of 32 different haplotypes, including 24 swine breeds and 5 Wild Boar populations. Figures on the internodes are bootstrap probabilities based on 1000 replications. Outgroup: *Phacochoerus africanus* (NC_008830).

The phylogenetic tree obtained based on the cytochrome b sequence highlighted two major clades: the European, containing the majority of the swine breeds from Europe, including the Mangalitsa breed and the Romanian Wild Boar, and the Asian comprising all the breeds from Asia, as well as some breeds from Europe, such as the Large White, Pietran, Berkshire or Spotted Black Jagubo. The Romanian and Italian Wild Boars form distinct clusters within the European clade, although this distribution is supported by a low bootstrap value (Figure 2). At the same time, all the haplotypes of the Mangalitsa breed, from both Romania and Hungary, were grouped together with other European breeds, in a distinct cluster.

**Figure 3:**
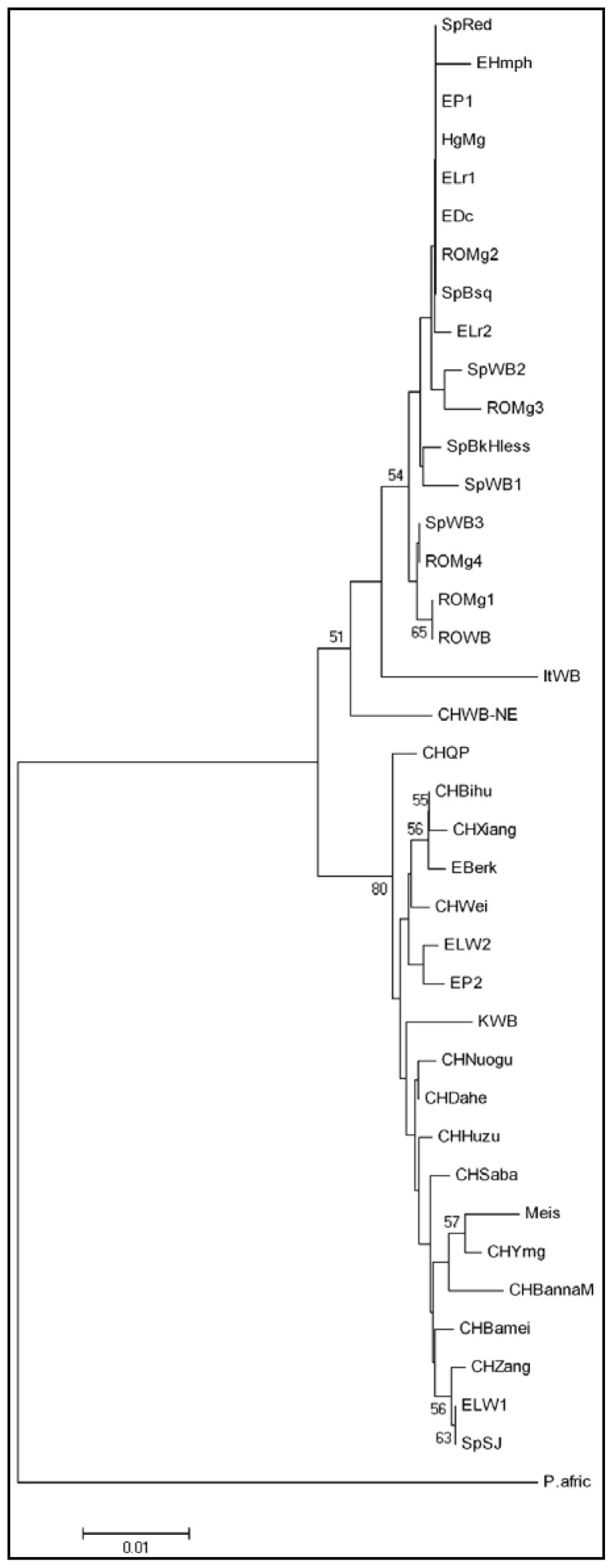
Neighbor-Joining tree based on D-loop sequence of 32 different haplotypes, including 24 swine breeds and 5 Wild Boar populations. Figures on the internodes are bootstrap probabilities based on 1000 replications. Outgroup: *Phacochoerus africanus* (NC_008830).

The tree obtained based on the D-loop fragment shows a slightly different topology compared to the tree obtained based on the cytochrome b gene (Figure 3). In this case, the two clades are not as clearly separated, since the North-East China Wild Boar is categorized as an isolated cluster at the level of the European clade. Within the European clade, the breeds are disposed in separate clusters and as a result the uniformity present in the tree constructed based on the cytochrome b gene disappears in this case. A haplotype of the Mangalitsa breed from Romania (ROMg1) appears in the same cluster with the Romanian Wild Boar, while two other haplotypes (ROMg3 and ROMg4) form separate branches together with the haplotypes of the Spanish Wild Boar. The Large White, Pietran, Berkshire and Spotted Black Jagubo breeds are also included in the Asian clade. Unfortunately, these phyologenetic relationships were supported by low bootstrap values.

**Figure 4:**
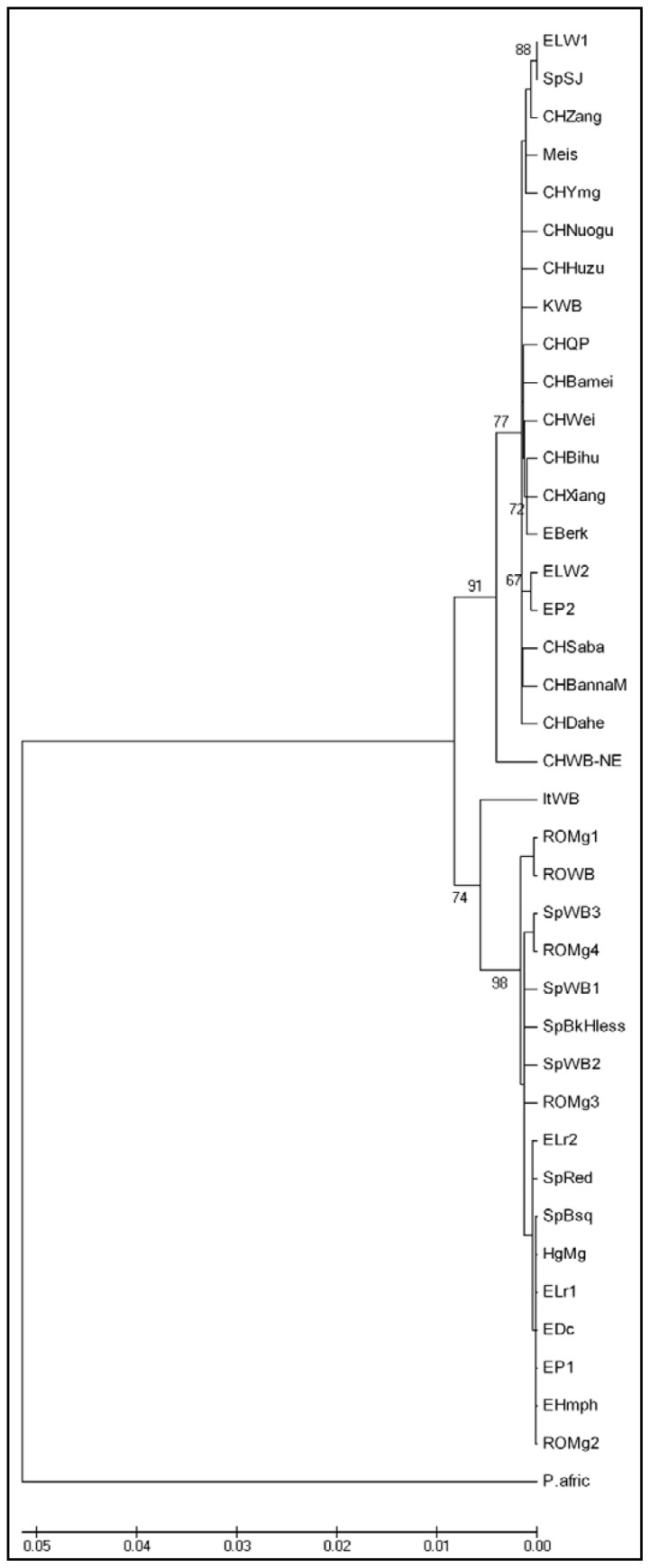
Neighbor-Joining tree based on D-loop and cytochrome b sequences. Figures on the internodes are bootstrap probabilities based on 1000 replications. Outgroup: *Phacochoerus africanus* (NC_008830).

Within the tree constructed based on the joint analysis of the two mitochondrial sequences (Figure 4) we can clearly notice only two clades, a European one and an Asian one. The North-East China Wild Boar is currently included, with a 91% bootstrap value, in the Asian clade. As expected, the European Large White, Pietran, Berkshire and Spotted Black Jagubo breeds still appear in the Asian clade. Their inclusion in this clade is not surprising and it was emphasized by other authors as well [17,18]. The presence in the Asian clade reflects the introduction of the Asian swine in Europe [19] and denotes the fact that European domestic breeds could have a more diverse genetic base than initially supposed. Consequently, it was suggested that swine breeds from East Asia, imported to England from the 17th to the 19th century, have contributed to the formation of the Large White and Berkshire breeds [20]. The inclusion of the Pietran breed in the Asian clade was emphasized by Alves *et al*., 2003 [18]. This affiliation can be correlated with the fact that the Large White and Berkshire specimens took part in the formation of the breed [19].

The four haplotypes of the Mangalitsa breed in Romania can be found in similar positions with those from the tree constructed based only on the fragment from the D-loop mitochondrial region. The exception is the ROMg2 haplotype that appears together with the Mangalitsa swine from Hungary in a separate cluster in the clade of the European breeds. The other three haplotypes appear to be clustered with those originating from the Wild Boars from Romania or Spain.

Thus, despite the low bootstrap support, the molecular data suggest that the Mangalitsa swine originates from primitive breeds that are probably derived directly from the Wild Boars in Central and Eastern Europe.

As far as the European Wild Boar is concerned, our results confirm previous studies undertaken on mtDNA sequences in domestic swine and boars [17,21] that have also shown that the Italian Wild Boar represents a distinct monophyletic group in the European clade. Kijas and Andersson, 2001 [22] have suggested that a distinct subspecies might have evolved in this region due to the isolation during the last glacial period. The results of our study are in agreement with this suggestion, as the Italian Wild Boar is clearly separate from the other European breeds.

Ultimately we calculated the net-average genetic distances between the European and the Asian clades (*K*) taking into account both the synonymous substitutions, as well as the non-synonymous ones, at the level of the cytochrome b gene. In the case of the D-loop region, the genetic distances were calculated separately for the two domains: ETAS and Central. The time period (*T*) that has elapsed since the separation of the two clades can be determined from the *T* = *K*/2*r* equation [23], where *r* represents the rate of nucleotide substitutions, a rate that was accurately determined by Pesole *et al*., 1999 [24], for the various regions at the level of the mammal mtDNA. The net-average genetic distances between the clades (expressed as divergence percentages) as well as the time period that has passed since the two clades were separated, is shown in Table 2.

Our study results suggest that the divergence between the European and the Asian clades occurred approximately 700,000 years ago. This assessment is in close agreement with the one obtained by Alves *et al*., 2003 [18], who stated that this divergence had occurred 780,000 years ago. Sbisa *et al*., 1997 [25] consider that the central domain of the D-loop region has great statistical fluctuations and thus will not always fit in the estimation of the divergence between the clades. Consequently, if we eliminate the very high value obtained for the central domain we will get an estimation of approximately 580,000 years, a value that is close to the value of 660,000 years achieved by Alves *et al*., 2003 [18].

## 3. Experimental Section

### 3.1. Sampling and DNA Extraction

Fresh blood samples from 45 Mangalitsa swine (“Suinprod” Farm, Roman, Romania) and 15 Wild Boars were harvested. The Mangalitsa specimens were chosen at random and closely related animals were avoided in the selection process. The isolation of genomic DNA from fresh blood was performed with Wizard Genomic DNA Extraction Kit (Promega) according to the manufacturer’s instructions.

### 3.2. DNA Amplification and Sequencing

The cytochrome b gene was amplified and sequenced using two pairs of primers: first pair (cytb1) amplified a fragment of 831 bp, and the second (cytb2) a fragment of 576 bp. The two amplified fragments were partially overlapped in the median area. The reactions were carried out in a 25 μL final volume containing PCR Buffer, 1.5 mM MgCl_2_, 200μM dNTP, 0.5 μM of each primer (cytbF1: 5′-ACCACGACCAATGACATGAA-3′; cytbR1: 5′-TGCTGGGGTGTAGTTGTCTG-3′; cytbF2: 5′-ACAACCCTACCGGAATCTCA-3′; cytbR2: 5′-GGCCCTCCTTTTCTGGTTTA-3′), 0.5 units of AmpliTaq Gold DNA Polymerase, DNA and nuclease-free water. PCR were performed using a 45 cycles thermocycler program with denaturation at 95 °C (30 s), annealing at 58 °C (30 s/cytb1), 61 °C (30 s/cytb2), respectively, and extension at 72 °C (60 s).

For the D-loop region, we have designed a pair of primers that amplified a fragment of 678 bp. The reactions were carried out in a 25 μL final volume containing PCR Buffer, 1.5 mM MgCl_2_, 200 μM dNTP, 0.5 μM of each primer (DLoopF: 5′-TTCGTATGCAAACCAAAACG-3′; DLoopR: 5′-TGTCCCGTAACCATTGACTG-3′), 0.5 units of AmpliTaq Gold DNA Polymerase, DNA and nuclease-free water. PCR was performed using a 45 cycles thermocycler program with denaturation at 95 °C (30 s), annealing at 58 °C (30 s) and extension at 72 °C (60 s).

PCR products were purified with the Wizard PCR Preps DNA Purification System Kit (Promega) and sequenced using the ABI Prism^®^BigDye Terminator Cycle Sequencing Reaction Kit (AppliedBiosystems) in an ABI Prism 3130 Genetic Analyzer. The sequencing reactions were performed both for forward and reverse strands. The sequences were processed using DNA Sequencing Analysis 5.1 Software (AppliedBiosystems), aligned with the BioEdit program [26] and refined manually.

### 3.3. Sequence Alignment and Molecular Phylogenetic Analysis

The final sequence for the cytochrome b gene was 1140 bp in length. The D-loop sequences were truncated to nps 15,455–16,068 to accommodate the short sequences already published (Table 3). The cytochrome b and D-loop sequences were aligned using the ClustalX 2.0.9 software [27], with a 10-gap opening penalty and a 0.10-gap extension penalty parameter, while the rest of the settings were kept as default. The genetic diversity in terms of number of haplotypes, haplotype and nucleotide diversity was estimated using DNAsp v5 [28]. In addition to our sequences representing different haplotypes in Mangalitsa and Wild Boar from Romania, mtDNA sequences available in GenBank were included in our study to ensure the best possible phylogenetic evaluation. The warthog (*Phacochoerus africanus*) was used as an outgroup species. The accession numbers and references for the published sequences are presented in Table 3.

The best-fit model of nucleotide substitution was selected with ModelTest [29]. A Neighbor-Joining phylogenetic tree was constructed on the basis of the Kimura 2-parameter distances [16] implemented in MEGA 4.0 [30]. Bootstrap analyses (1000 replications) were used to assess the confidence of each node. The GenBank sequence with the accession number AJ002189 [15] was used as reference and a basis for the sequence numbering.

## 4. Conclusions

This is the first study focusing on the phylogenetic relationships of the Mangalitsa breed and other swine breeds from Europe and Asia.

The results obtained establish the influence of the Asian breeds on the following European breeds: Large White, Berkshire, Spotted Black Jagubo and Pietran. The Mangalitsa breed was included in the European breed cluster, while three out of the four haplotypes discovered were associated with separate clusters that include the Spanish or Romanian Wild Boars. The results of our study show a remarkable closeness of the Mangalitsa breed to the European Wild Boars, a closeness that was also confirmed by phenotypic similarities including the presence of fur on the entire body. The primitive character of this breed is also emphasized by its distribution in monophyletic groups with European Wild Boars and other primitive breeds.

As far as the divergence between the European and the Asian clades is concerned, the results we obtained, based on the data resulting from the cytochrome b gene sequences and the ETAS domain at the level of the D-loop region, suggest that it occurred approximately 580,000 years ago. This divergence supports the hypothesis of a multiple and independent domestication process that occurred a long time after the divergence of the two clades. This hypothesis is also supported by other studies [34,39].

## Figures and Tables

**Table 1:** Total number of haplotypes and polymorphic sites with their dispersion within 24 different swine breeds and 5 Wild Boar populations.

Breed name	Polymorphic sites	Haplotypes	Transitions	Transversions	Deletions
*European breeds*					

Mangalitsa (Romania)	9	4	8	1	-
Mangalitsa (Hungary)	3	1	2	1	-
Black Hairless	3	1	3	-	-
Red	4	1	3	1	-
Duroc	3	1	2	1	-
Large White	33	2	32	-	1
Landrace	4	2	4	-	-
Pietran	32	2	31	-	1
Hampshire	6	1	4	2	-
Spotted Black Jabugo	29	1	28	-	1
Basque	3	1	2	1	-
Berkshire	33	1	30	2	1

*Asiatic breeds*					

Nuogu	31	1	30	-	1
Banna Mini	32	1	31	-	1
Dahe	26	1	25	-	1
Saba	30	1	29	-	1
Zang	31	1	30	-	1
Wei	27	1	26	-	1
Qing Ping	27	1	26	-	1
Bamei	29	1	28	-	1
Huzu	29	1	28	-	1
Yimenghei	32	1	31	-	1
Bihu	28	1	27	-	1
Xiang	28	1	27	-	1
Meishan	36	1	32	3	1

*Asiatic and European Wild Boars*					

Wild Boar (N-E China)	26	1	25	-	1
Wild Boar (Koreea)	32	1	30	1	1
Wild Boar (Italy)	24	1	22	2	-
Wild Boar (Romania)	2	1	2	-	-
Wild Boar (Spain)	11	3	10	1	-

**Table 2:** Net-average genetic distances between clades (*K*) and the time period that had passed since the separation of the two clades (*T*).

mtDNA region	Nucleotide substitution rate (%) [24]	*K* ± SD (%)	*T* (× 10^3^ years)
*Cytochrome b gene*			

Synonymous positions	27.4 ± 3.3	3.875 ± 0.011	707
Non-synonymous positions	1.8 ± 0.3	0.154 ± 0.001	428

*D-loop region*			

ETAS Domain	19.4 ± 7.8	2.273 ± 0.006	586
Central Domain	3.8 ± 1.9	0.843 ± 0.004	1109

Average (including Central Domain)			707

Average (without Central Domain)			574

**Table 3:** D-loop and cytochrome b sequences from pigs of several breeds obtained from GenBank and used in this study.

Abbreviation	Accession number	Breed/Location	References
SpBkHless	AY237494/cytb AY232852/D-loop	Black Hairless/Spain/Europe	Alves *et al.*, 2003 [18]
SpRed	AY237498/cytb AY232856/D-loop	Red/Spain/Europe	Alves *et al.*, 2003 [18]
SpWB1	AY237510/cytb AY232868/D-loop	Wild Boar 1/Spain/Europe	Alves *et al.*, 2003 [18]
SpWB2	AY237513/cytb AY232871/D-loop	Wild Boar 2/Spain/Europe	Alves *et al.*, 2003 [18]
SpWB3	AY237515/cytb AY232873/D-loop	Wild Boar 3/Spain/Europe	Alves *et al.*, 2003 [18]
EDc	AY237519/cytb AY232877/D-loop	Duroc/Europe	Alves *et al.*, 2003 [18]
ELW1	AY237524/cytb AY232882/D-loop	Large White 1/Europe	Alves *et al.*, 2003 [18]
ELW2	AY237525/cytb AY232883/D-loop	Large White 2/Europe	Alves *et al.*, 2003 [18]
ELr1	AY237526/cytb AY232884/D-loop	Landrace 1/Europe	Alves *et al.*, 2003 [18]
ELr2	AY237527/cytb AY232885/D-loop	Landrace 2/Europe	Alves *et al.*, 2003 [18]
EP1	AY237528/citb AY232886/D-loop	Pietran 1/Europe	Alves *et al.*, 2003 [18]
EP2	AY237529/cytb AY232887/D-loop	Pietran 2/Europe	Alves *et al.*, 2003 [18]
Meis	AY237530/cytb AY232888/D-loop	Meishan/China	Alves *et al.*, 2003 [18]
SpSJ	AY237532/cytb AY232890/D-loop	Spotted Black Jabugo/Spain/Europe	Alves *et al.*, 2003 [18]
SpBsq	AY237533/cytb AY232891/D-loop	Basque/Spain/Europe	Alves *et al.*, 2003 [18]
HgMg	AY237534/cytb AY232892/D-loop	Mangalitsa/Hungary/urope	Alves *et al.*, 2003 [18]
CHNuogu	DQ466081	Nuogu/China	Wang *et al*., 2006 [31]
CHBannaM	GQ220328	Banna Mini/China	Su *et al*., 2009 [32]
CHDahe	GQ220329	Dahe/China	Su *et al*., 2009 [33]
CHSaba	EF545567	Saba/China	Wu *et al*., 2007 [34]
CHZang	EF545576	Zang/China	Wu *et al*., 2007 [34]
CHWei	EF545577	Wei/China	Wu *et al*., 2007 [34]
CHQP	EF545582	Qing Ping/China	Wu *et al*., 2007 [34]
CHBamei	EF545583	Bamei/China	Wu *et al*., 2007 [34]
CHHuzu	EF545588	Huzu/China	Wu *et al*., 2007 [34]
CHYmg	EF545589	Yimenghei/China	Wu *et al*., 2007 [34]
CHBihu	EF545591	Bihu/China	Wu *et al*., 2007 [34]
CHXiang	EF545593	Xiang/China	Wu *et al*., 2007 [34]
KWB	EU090703	Wild Boar/Korea	Cho *et al*., 2007 [35]
CHWB-NE	EU333163	Wild Boar/Nord-East China	Yu *et al*., 2007 [36]
Eberk	AY574045	Berkshire/Europe	Cho *et al*., 2004 [37]
EHmph	AY574046	Hampshire/Europe	Cho *et al*., 2004 [38]
ItWB	AF304201	Wild Boar/Italy/Europe	Kijas and Andersson, 2001 [22]
P. afric	NC_008830	*Phacochoerus africanus*	Wu *et al*., 2007 [34]
